# Clinical Applications of Mixed Reality and 3D Printing in Congenital Heart Disease

**DOI:** 10.3390/biom12111548

**Published:** 2022-10-24

**Authors:** Ivan Lau, Ashu Gupta, Abdul Ihdayhid, Zhonghua Sun

**Affiliations:** 1 Discipline of Medical Radiation Science, Curtin Medical School, Curtin University, Perth, WA 6845, Australia; 2 Department of Medical Imaging, Fiona Stanley Hospital, Perth, WA 6150, Australia; 3 Curtin Medical School, Faculty of Health Sciences, Curtin University, Perth, WA 6845, Australia; 4 Department of Cardiology, Fiona Stanley Hospital, Perth, WA 6150, Australia

**Keywords:** 3D printing, extended reality, mixed reality, congenital heart disease, diagnosis, visualization

## Abstract

Understanding the anatomical features and generation of realistic three-dimensional (3D) visualization of congenital heart disease (CHD) is always challenging due to the complexity and wide spectrum of CHD. Emerging technologies, including 3D printing and mixed reality (MR), have the potential to overcome these limitations based on 2D and 3D reconstructions of the standard DICOM (Digital Imaging and Communications in Medicine) images. However, very little research has been conducted with regard to the clinical value of these two novel technologies in CHD. This study aims to investigate the usefulness and clinical value of MR and 3D printing in assisting diagnosis, medical education, pre-operative planning, and intraoperative guidance of CHD surgeries through evaluations from a group of cardiac specialists and physicians. Two cardiac computed tomography angiography scans that demonstrate CHD of different complexities (atrial septal defect and double outlet right ventricle) were selected and converted into 3D-printed heart models (3DPHM) and MR models. Thirty-four cardiac specialists and physicians were recruited. The results showed that the MR models were ranked as the best modality amongst the three, and were significantly better than DICOM images in demonstrating complex CHD lesions (mean difference (MD) = 0.76, *p* = 0.01), in enhancing depth perception (MD = 1.09, *p* = 0.00), in portraying spatial relationship between cardiac structures (MD = 1.15, *p* = 0.00), as a learning tool of the pathology (MD = 0.91, *p* = 0.00), and in facilitating pre-operative planning (MD = 0.87, *p* = 0.02). The 3DPHM were ranked as the best modality and significantly better than DICOM images in facilitating communication with patients (MD = 0.99, *p* = 0.00). In conclusion, both MR models and 3DPHM have their own strengths in different aspects, and they are superior to standard DICOM images in the visualization and management of CHD.

## 1. Introduction

Congenital heart disease (CHD) is considered one of the most challenging pathologies to manage in clinical practice due to its broad spectrum of morphologies that vary from individual to individual [1,2,3]. If surgery or intervention is required to repair the heart lesion, a full understanding of the anomalous cardiac structure plays a fundamental role in a successful surgery [3,4]. However, current visualization techniques based on cardiac computed tomography (CT) or magnetic resonance imaging with volume rendering lack realism as they do not depict the actual depth of the object [1,2].

Three-dimensional (3D) printing is a technique that has been adopted in cardiovascular medicine in the last two decades to demonstrate the geometric relationships between the intra- and extra-cardiac structures [5,6,7,8,9,10,11,12,13,14]. One recent study presented the use of 3D-printed heart models (3DPHM) in 40 cases where types of surgical strategy could not be determined from conventional imaging. 3DPHM helped the surgical team to decide and modify the best treatment plan for 31 cases, while the remaining nine cases remained equivocal [15]. Other studies have also demonstrated the usefulness of 3DPHM in surgical simulation, hands-on training, and medical education [3,5,6,7,8,9,10,11,12,13,14,15,16,17,18]. Despite these benefits of using 3DPHM, the time and cost of producing them are the two main factors that impede its widespread application in the medical field [3]. Furthermore, as it is a relatively novel technology for medical applications, its application still requires a long process of standardization and quality control [3].

Mixed reality (MR), which is an advancement of augmented reality (AR), has only very recently been introduced to the realm of medicine [19]. MR works by overlaying virtual objects in real-world settings via head-mounted displays or hand-held mobile devices, allowing the users to manipulate or interact with the virtual objects within an immersive environment [20]. A recent study by Gehrsitz et al. has demonstrated the usefulness of MR in the pre-operative planning of pediatric heart surgeries. The use of MR was found to significantly improve the depth perception and portrayal of the pathology when compared to the two-dimensional (2D) medical images and 3DPHM [21]. The use of AR has also been shown constructive in Valve-in-Valve Transcatheter Heart Valve Implantation and percutaneous coronary intervention procedures [22,23]. The superimposition of the computed tomography angiography (CTA) reconstructions with the real-time fluoroscopic images allowed the cardiologist to accurately deploy the transcatheter heart valve with minimal contrast administration [22]. However, both the use of MR in medicine and the application of 3DPHM in complex cardiac surgeries are still in their infancy. Especially for MR, its superiority over the current visualization technique requires further validation. The published article to compare 3D printing and MR in pediatric heart surgeries is limited to one surgeon’s assessment based on a single-center experience [21].

Hence, the aim of this study is to investigate the clinical value of both MR and 3D printing in CHD by comparing these innovative technologies concurrently with the conventional visualization technique in terms of assisting clinical diagnosis, medical education, pre-operative planning, and intraoperative guidance of the CHD surgeries through evaluations from cardiac specialists and physicians.

## 2. Materials and Methods

This is a cross-sectional study performed to assess the cardiac specialists’ and physicians’ opinions on the two rapidly evolving 3D visualization techniques: 3D printing and MR, compared to the conventional method using Digital Imaging and Communications in Medicine (DICOM) images. This study was conducted following the clinical practice guidelines with ethics approval sought from Curtin University Human Research Ethics Committee.

### 2.1. Generation of the Digital Heart Models

Two cases of anonymized cardiac CT angiography scans featuring CHD were retrospectively chosen as the source data. In order to find out if the study result is dependent on the complexity of the disease, we included cases with Aristotle Basic Complexity Level (ABCL) of 1 and 4: atrial septal defect (ASD) and Double Outlet Right Ventricle (DORV), respectively. The CT datasets were transferred to a workstation for image post-processing using Mimics Innovation Suite 22.0 (Materialise HQ, Leuven, Belgium). The segmentation process was semi-automated using a thresholding technique by selecting the blood pool as area of interest. After removing all the unwanted structures, a 1 mm thick shell was added to the models using 3-Matic (Materialise HQ, Leuven, Belgium). Then, the digital model was hollowed out and smoothed. The digital heart models took approximately 30 min each to be generated.

### 2.2. Three-Dimensional Printing

**Figure 1:**
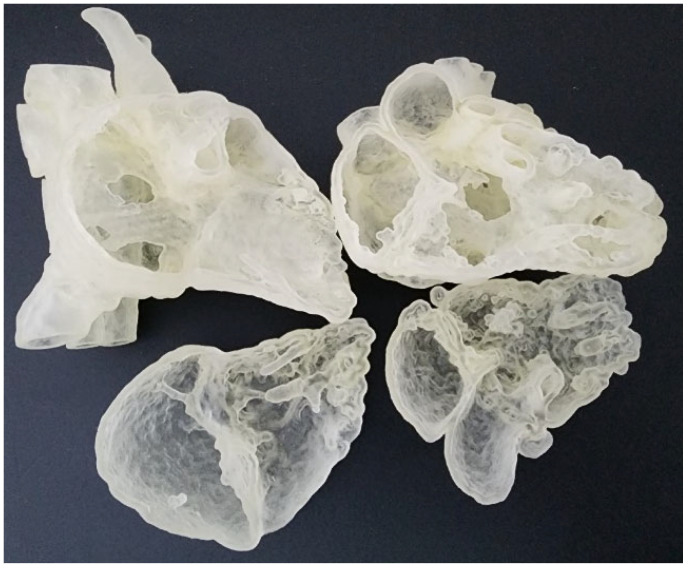
Flexible heart models printed in Agilus 30 featuring atrial septal defect (**left column**) and double outlet right ventricle (**right column**).

The digital models were exported in standard tessellation language (STL) format for 3D printing. In order to view the intra-cardiac structures, the STL files were imported to Meshmixer (Autodesk, San Rafael, CA, USA) for the separation of the heart models into two compartments. A suitable cutting plane transecting the right atrium and right ventricle was created on both models. They were then exported as separate STL files for 3D printing. They were printed commercially in clear Agilus 30 (Objective 3D, Stratasys, Melbourne, Victoria, Australia), which has a shore hardness of 30A and therefore are able to provide a flexible touch (Figure 1). The ASD model costs AUD350, whereas the DORV costs AUD270 to print.

### 2.3. Development of Mixed Reality Application

**Figure 2:**
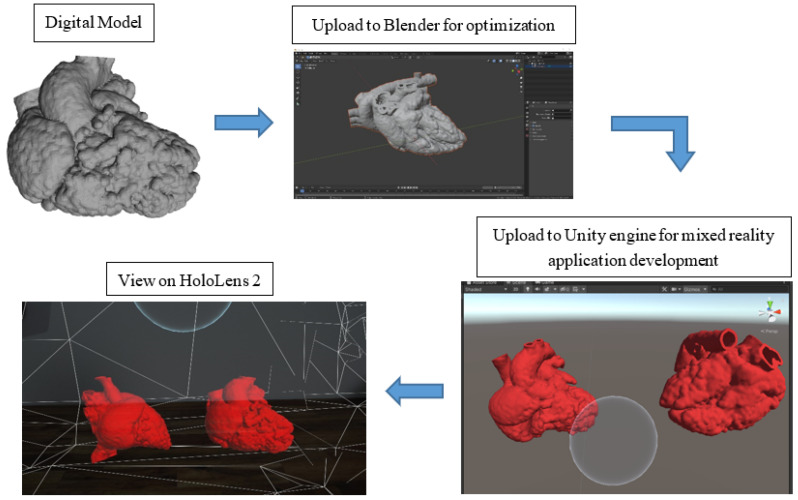
A flow diagram illustrating the process of mixed reality application development.

Figure 2 illustrates the process of the development of the MR application. The digital models were exported in object file (OBJ) format and loaded into Blender (V2.91.0, Blender Foundation, Amsterdam, The Netherlands) for optimization for holographic viewing. Due to the highly complex polygon mesh of the heart models, the models were required to undergo primitive UV sphere in Blender to optimize the performance of the application on HoloLens 2 (Microsoft Crop., Redmond, DC, USA). For the development of the MR application, Unity engine (V2020.3.13f1, Unity Technologies ApS., San Francisco, CA, USA), Microsoft OpenXR Plugin, Mixed Reality Toolkit (MRTK) (Version 2.7.0, Redmond, DC, USA), and Microsoft Visual Studio 2019 (Version 16.11.9, Microsoft Corp., Redmond, DC, USA) were utilized. The initial steps to set up the application were referenced from the Microsoft HoloLens 2 tutorial webpage [24].

**Figure 3:**
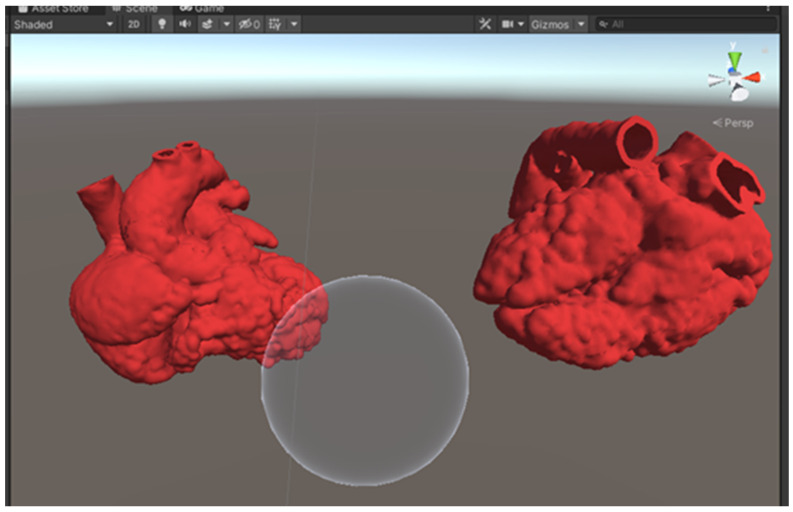
A screenshot of the Unity scene with 2 heart models and a sphere to serve as the clipping tool.

**Figure 4:**
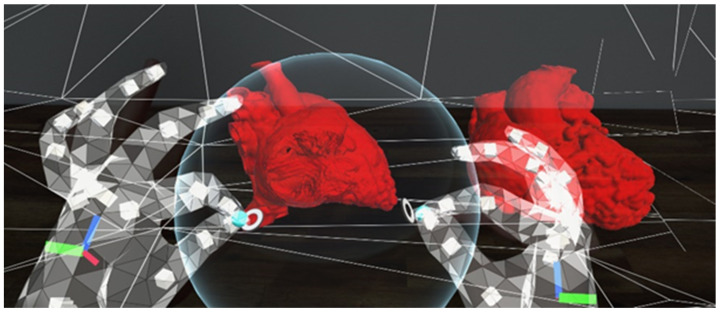
A screenshot using the HoloLens 2. The user is using the sphere to cut through the heart models in order to view the intra-cardiac structures. The sphere can be enlarged or sized down to change the amount of anatomy to be cut out.

Within Unity, both optimized models were loaded into the scene. The red color was added as the models’ material to give the heart models a more realistic look. A sphere was added to the scene with a custom shader script written in High-Level Shading Language (HLSL), allowing the sphere to serve as a clipping tool (Figure 3). A C# script was also written to allow the clipping range to change according to the size of the sphere, which is determined by the users. This allows the users to manipulate the cutting plane of the MR heart models by changing the size of the sphere using different hand gestures (Figure 4). A video of the MR application can be viewed in Supplementary File S1.

### 2.4. Participant Recruitment and Data Collection

Thirty-four cardiac specialists and physicians were recruited from public and private hospitals in Western Australia. Each participant attended a one-to-one session with one of the authors (I.L); therefore, their responses were independent of each other. Each participant went through 3 different stations for assessment of heart models in different forms. In order to mimic the real clinical situation, the participants were not informed of the diagnosis during the assessment and were asked to make their own diagnosis. The answers were only revealed at the end of the third station. In order to keep the amount of time each participant spent on assessing the models consistent, each participant was given a time limit of 3 min per station.

**Figure 5:**
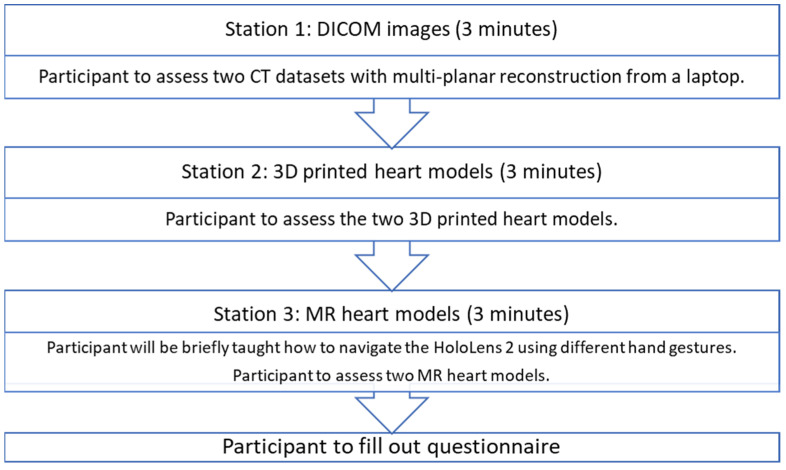
The process each participant undertook.

At station 1, the participants were presented with the CT images of ASD and DORV cases on a laptop using an open-source DICOM viewer, RadiAnt (Medixant, Poznan, Poland). Without being told the diagnosis of the cases, they were asked to examine the anatomy and pathology based on the DICOM datasets. As this is the routine approach to examining the pathologies, this station acted as a control for the study. At station 2, the participants were presented with the 3DPHM. At station 3, the participants were presented with HoloLens 2. They were first given brief instructions on how to manipulate the MR heart models using different hand gestures (zooming in and out, changing the clipping plane, rotating the models) prior to the assessment of the models. The time taken for each participant to learn to manipulate the MR models was excluded from the 3-minute time limit. After that, the participants filled out a questionnaire to rank each modality from 1 to 3 for each of the questions. The questionnaire was designed particularly to focus on (i) the ability of each modality to display normal/abnormal cardiac and vascular structures and pathologies; (ii) the utility of each modality in educating young doctors or physicians about CHD; (iii) the usefulness of each modality in pre-operative planning and intraoperative guidance of CHD surgeries. For participants who are non-surgical nor interventional, their questionnaire only focused on (i) and (ii). The questionnaire can be found in Supplementary File S2. Figure 5 illustrates this process.

### 2.5. Statistical Analysis

The quantitative data from the questionnaire were analyzed using IBM SPSS statistical package, version 26 (IBM Corp, Armonk, NY, USA). The normality of the data was assessed by a normal probability plot, and skewness and kurtosis of the distribution were reported. To assess the statistical differences in the mean scores between DICOM images, 3DPHM, and the MR models, a general linear model was applied with gender, age, occupation, AR experience, and 3D-printing experience as confounding factors. Independent samples t-test was used to compare the responses between interventionists and non-interventionists. A *p*-value of <0.05 is considered statistically significant. The qualitative data from the free-text section of the questionnaire were analyzed using thematic analysis.

## 3. Results

Out of the 34 participants, 27 were male, and 16 were below the age of 40. There were 8 cardiac surgeons, 16 interventional cardiologists and cardiology registrars, 6 cardiologists and cardiac imaging fellows, and 4 radiologists and general physicians (Table 1). About 58.8% and 52.9% of the participants indicated they had never used AR and 3D printing in their medical practice, respectively.

The responses for each question on the questionnaire follow approximately a normal distribution, except for Question 7 with the modality of 3D printing (skewness = 2.986 for ASD, 2.728 for DORV). The results of the normality of the data can be found in Supplementary File S3. Table 2 presents the mean rank of each modality for each question. The participants were asked to rank the modality from 1 to 3 (1 being the best modality). Therefore, the closer the mean rank gets to 1, the better the modality is perceived by the participants. Table 3 presents the mean rank differences between modalities and their respective *p*-values. Table 4 presents the mean differences in the responses between the two occupation groups, interventionalists and non-interventionalists. The result shows that there are no significant differences in their responses.

The 3DPHM were ranked as the best modality to visualize the heart defect for ASD, and the MR model as the best for DORV. However, a significant difference is only found between the mean rank of DICOM and MR for DORV (*p* = 0.01). In other words, the participants found that for a more complex type of CHD, the MR models allowed them to appreciate the heart defects better.

MR models were ranked the best in demonstrating the spatial relationship between the cardiac structures for both types of CHD. It was found to be significantly better compared to DICOM for DORV (*p* = 0.00). Similarly, MR models were ranked the best for depth perception for both CHD. It is significantly different from DICOM (*p* = 0.00 for both ASD and DORV). The 3DPHM, which was ranked second for depth perception, is also significantly different from DICOM (*p* = 0.02 for ASD).

As for the learning of the CHD pathology, MR models were ranked the best, and it is found to be significantly better than the DICOM images (*p* = 0.00 for both ASD and DORV). 3DPHM, which were ranked second in this category, also achieved statistical significance compared to DICOM (*p* = 0.02 for ASD).

When it comes to communication with patients, 3DPHM were ranked as the best modality for both CHD. The pairwise comparisons indicate that 3DPHM were significantly different from both DICOM and MR models (*p* = 0.00 for both ASD and DORV). In fact, nearly 90% (30 out of 34) of participants indicated their preference for using 3DPHM as a communication tool with patients. This explains why the response to this particular question is greatly skewed.

MR models were also ranked the most superior in helping the participants to foresee possible complications associated with the surgeries or interventions, especially for complex CHD such as DORV (*p* = 0.05) (Table 3). For both types of CHD, MR models were ranked as the best tool for pre-operative planning. Its’ mean rank is significantly different from DICOM (*p* = 0.00 for ASD, *p* = 0.02 for DORV). Generally speaking, the complexity of CHD does not cause a significant difference in the results (Supplementary File S4).

For the free-text section of the questionnaires, the participants’ feedback can be categorized into five themes: (i) intuitiveness of the clipping tool in the MR application; (ii) requirement of training for MR application; (iii) advantages of MR application; (iv) limitations of MR application; and (v) suggestions for MR application (Table 5). There were more participants who found that the MR application was easy to use (*n* = 10) compared to those who indicated that it was not fully intuitive (*n* = 4) and had a steep learning curve for them (*n* = 2). Nine participants commented that more training is required for them to get used to the MR application. Seven participants mentioned the usefulness of the clipping tool in visualizing the internal cardiac structures at different angles, which is difficult to achieve on DICOM. Despite so, the clipping tool does have a limitation of creating artificial defects on the MR models due to its shape of a 3D sphere (*n* = 2). Thus, one of the participants suggested replacing the clipping tool with a flat 2D plane. More feedback examples from participants are detailed in Table 5.

## 4. Discussion

Visualization of the anatomical features of the diseased heart plays a vital role in the success of CHD surgeries. By improving the degree of verisimilitude of the visualization technique, the cognitive gap between the 2D medical images and the real heart in 3D can be closed, hence allowing the surgeons to decide the best surgical approach [25]. To date, there have been increasing reports on the usefulness of 3DPHM in redefining the best surgical strategies for complex CHD, in facilitating communication with patients, and in medical education for healthcare workers and students [1,2,4,5,6,7,8,9,10,11,12,13,14,15,16,17,18]. With the advancement in high-quality holographic visualization, the application of MR in the medical field has also been explored in recent years for its use in pre-operative planning [19,20,21,22,23,25,26,27].

To the best of our knowledge, this cross-sectional study is the first in the literature to concurrently compare the usefulness of these two emerging technologies to the conventional visualization method in the clinical management of CHD. The evaluations from the cardiac specialists and the physicians based on the two provided CHD cases suggested promising results for both of these technologies. This is especially apparent for MR, which was ranked as the best modality for most of the questions. In real clinical applications, the potential benefits of MR heart models in improving the visualization of the pathology, medical education, and pre-operative planning should not be overlooked. In terms of communication facilitation, 3DPHM is the best approach according to the results, as it is tangible and is able to effectively demonstrate the spatial relationship between the cardiac structures in 3D.

Even though the participants did not find the MR models as useful as 3DPHM in facilitating communication among the health professionals in the present study, its usefulness in this aspect should not be underestimated. The study by Kumar et al. reported that MR is extremely useful for multidisciplinary team meetings. All the surgeons wore the HoloLens headset and were able to view and interact with the heart and liver models in the same 3D space. This facilitated the discussion among the surgeons to decide the best surgical approach [26]. We believe the main reason behind this difference in study findings is that there was only one HoloLens 2 headset for use in our study. Therefore, the evaluations were strictly limited to singer-user experience instead of multi-user experience.

Nonetheless, the other findings about MR from our study are similar to other studies [25,27]. Ye et al. reported the value of using HoloLens in shortening the time taken for pre-operative planning of DORV surgeries and improving the accuracy of the selection of surgical approach [25]. The results of our study also suggested MR is the best modality to aid in pre-operative planning. In another study by Brun et al., the pediatric heart team members rated highly positively of the MR models in terms of depth perception, morphology understanding, and the ability of it to share the view of the heart holograms [27]. In our study, we have provided more insights by comparing MR with 3DPHM and DICOM, and the results show that MR is much better than the others in demonstrating heart defects, depth information, and spatial relationship.

Compared to 3D printing, one of the advantages of MR is the avoidance of 3D-printing turnaround time, which is usually long [25,27]. Therefore, for complex emergency cases when an urgent surgical approach decision is required, MR is a better option. On the other hand, unlike 3DPHM, which can only demonstrate a single cutting plane [25], the MR models can demonstrate unlimited numbers of cutting plane at a user-defined angle or perspective and, therefore, greatly improve the perception of the anatomy [27]. Further, there will be an additional cost associated with 3D printing each time a model is printed. In this aspect, MR could be more cost-effective depending on the departments’ needs.

Despite these advantages, MR does come with its own limitation. In order to exploit the full benefits of MR, the users will have to spend some time training to get used to manipulating the MR models. This is the main concern that the participants indicated in the questionnaire (Table 5). In fact, during the face-to-face session, the author noticed that the learning curve for each participant to master a skill varied differently. Participants with younger age (in their 30s) tend to learn the gesturing techniques quicker and, therefore, are able to manipulate the MR models better compared to the others, despite the absence of previous experience with AR. Another potential issue associated with MR or extended reality headsets is motion sickness. By default, the MRTK on Unity, which was used to build the application, has a spatial awareness system enabled. It creates meshes of triangles of the real-world surfaces (Figure 3) to allow interactions of the holograms with the real environment [28]. Due to this, a number of participants complained about motion sickness after taking the headset off, as the meshes of triangles constantly change with the users’ head movement. For the subsequent meetings with other participants, the setting was changed so that the triangle meshes would be hidden, and no more complaints were made about motion sickness. Future studies should take note of this as it is related to work health and safety issues if it were to be used intra-operatively/during interventions. Another limitation of HoloLens 2 is that the image contrast and the sensitivity of the hand tracking are very much dependent on the room lighting. If the room is too dark or bright, the quality of both of the aforementioned elements will be degraded, hence affecting the users’ experience.

This study has a few limitations. First, as the participants were asked to assess the same two CHD cases in different modalities, by the time they got to station 3, they already more or less knew the pathomorphology of the CHD, and hence the results might be subjected to bias from repeated measures. In order to prevent this, future studies could randomize the sequence of the modalities to different participants. Second, the time limit of 3 min per station might not be enough for some to evaluate the modality in a comprehensive manner. This might have introduced some bias to the participants’ responses. Third, even though a brief tutorial was already given to the participants in maneuvering the MR models, some of them still struggled to do so. This might have affected their experience during the assessment of the MR models, impeding them from evaluating the models properly. Future studies could address this limitation by running a simulation tutorial within the MR headset, very much like a mini game, to serve as guidance for the participants. Fourth, we only chose two CHD cases in this study. Although they represented simple and complex CHD situations, more cases with different pathologies would be desirable to allow robust conclusions to be drawn. In spite of this limitation, our models were printed with the Agilus A30 material simulating normal cardiac tissue properties. Recent studies have shown the value of using Agilus A30 to print an aortic dissection model for the investigation of optimal CT angiography protocols [29,30]. Current literature shows a wide range of materials (from plastic to polylactic acid and thermoplastic polyurethane to rigid materials such as resin) and printers being used for 3D-printing CHD models, as indicated by a recent review article [31]. These 3D-printed CHD models are acceptable for education purposes due to their high accuracy in replicating both normal anatomy and pathology (the mean dimensional difference between 3D-printed models and original source images is <0.5 mm) [2,12,14,32,33,34]. However, when used for pre-surgical planning and simulation purpose, a more realistic model (soft and elastic) is preferred by clinicians as it allows the user to acquire tactile experience when performing cardiac or interventional procedures [35,36]. This was not assessed in this study as we did not focus on the user’s experience of using 3DPHM for simulations. Identification of the ideal 3D-printed heart models by clinicians with regard to the model’s resilience, toughness, and hardness deserves to be investigated. Further, the use of 3DPHM or MR to contribute to patient treatment and outcomes will need to be investigated in further studies to determine how these technologies advance clinical practice.

Finally, the locally developed MR application in this study is very basic, without the menu bar on the interface. Despite this, the participants already had very positive comments about this tool. In the future, we would like to further develop this application by adding some interactive buttons that would allow the users to show or hide certain structures, measure, and to auto-crop the models in different axes. Another 3D visualization tool is generation of 3D Portable Document Format (PDF) with embedded 3D objects (segmented volume data), which offers free rotatability and slicing features, thus allowing interactive visualization of anatomical structures [37,38]. Comparison of 3D PDF with MR in CHD could reveal the real benefits of virtual reality in clinical practice, and this could be considered in future studies.

## 5. Conclusions

This study has shown the key findings that the MR models were ranked as the preferred tool in demonstrating complex CHD lesions, enhancing depth perception, portraying the spatial relationship between cardiac structures, as a learning tool of the pathology, and facilitating pre-operative planning. The 3DPHM were ranked the preferred tool in facilitating communication with patients, enhancing depth perception, and as a learning tool of the pathology. Both MR and 3DPHM serve as complementary tools to the current image visualization method by providing more valuable information beneficial to diagnostic assessment of patients with CHD.

## Figures and Tables

**Table 1:** Characteristics of study participants.

Variables	No. of Participants (%)
*Gender*	
Male	27 (79.4)
Female	7 (20.6)
*Age*	
Below 40	16 (47.1)
Above 40	15 (44.1)
Missed responses	3 (8.8)
*Occupation*	
*Surgical/interventional*	
Cardiac surgeon	8 (23.5)
Interventional cardiologist, cardiology registrar	16 (47.0)
*Non-surgical/non-interventional*	
Cardiologist, cardiac imaging fellow	6 (17.6)
Radiologist, general physicians	4 (11.8)
*AR experience*	
Yes	12 (35.3)
No	20 (58.8)
Missed responses	2 (5.9)
*3D-printing experience*	
Yes	14 (41.2)
No	18 (52.9)
Missed responses	2 (5.9)

3D, three-dimensional; AR, augmented reality.

**Table 2:** The mean rank of different modalities for each question.

Questions	Modality	Atrial Septal Defect			Double Outlet Right Ventricle		
		Mean	SD	*p*-Value	Mean	SD	*p*-Value
1. Assessment of major vessels	DICOM	1.85	0.86	0.28	2.09	0.90	0.85
	3DPHM	2.03	0.67		2.06	0.69	
	MR	2.12	0.91		1.85	0.86	
2. Appreciation of heart defects	DICOM	2.47	0.83	0.05	2.50	0.75	0.05
	3DPHM	1.62	0.74		1.76	0.74	
	MR	1.91	0.67		1.74	0.75	
3. Spatial relationship between the cardiac structures	DICOM	2.56	0.75	0.02	2.65	0.69	0.00
	3DPHM	1.74	0.71		1.85	0.74	
	MR	1.71	0.72		1.50	0.56	
4. Depth perception	DICOM	2.68	0.68	0.00	2.62	0.70	0.00
	3DPHM	1.74	0.67		1.85	0.70	
	MR	1.59	0.66		1.53	0.66	
5. Pathology learning	DICOM	2.59	0.74	0.00	2.50	0.79	0.01
	3DPHM	1.74	0.50		1.88	0.73	
	MR	1.65	0.65		1.59	0.66	
6. Communication tool with another health professional	DICOM	2.00	0.89	0.09	2.15	0.86	0.33
	3DPHM	1.79	0.73		1.76	0.78	
	MR	2.21	0.81		2.09	0.79	
7. Communication tool with patients	DICOM	2.59	0.61	0.00	2.65	0.54	0.00
	3DPHM	1.18	0.52		1.21	0.59	
	MR	2.24	0.55		2.15	0.56	
8. Prepares me for surgery/intervention	DICOM	2.23	0.87	0.18	2.27	0.83	0.09
	3DPHM	2.00	0.69		2.14	0.77	
	MR	1.77	0.87		1.59	0.73	
9. Helps to understand possible complications	DICOM	2.22	0.85	0.92	2.39	0.78	0.05
	3DPHM	1.91	0.73		2.00	0.80	
	MR	1.87	0.87		1.61	0.72	
10. Pre-operative planning	DICOM	2.43	0.79	0.03	2.39	0.78	0.03
	3DPHM	1.87	0.76		1.77	0.73	
	MR	1.70	0.77		1.52	0.73	
11. Intra-operative guidance	DICOM	2.39	0.78	0.39	2.39	0.78	0.06
	3DPHM	1.91	0.73		2.04	0.77	
	MR	1.70	0.82		1.57	0.73	

3DPHM, three-dimensional printed heart models; DICOM, Digital Imaging and Communications in Medicine; MR, mixed reality; SD, standard deviation.

**Table 3:** Mean differences between modalities and significance values of pairwise comparisons.

Questions		Atrial Septal Defect			Double Outlet Right Ventricle		
		Mean Diff.	SD	*p*-Value ^a^	Mean Diff.	SD	*p*-Value ^a^
1. Assessment of major vessels	DICOM-3DPHM	−0.18	1.24	0.41	0.03	1.36	1.00
	DICOM-MR	−0.26	1.64	0.15	0.24	1.62	1.00
	3DPHM-MR	−0.09	1.36	1.00	0.21	1.27	1.00
2. Appreciation of heart defects	DICOM-3DPHM	0.85	1.42	0.06	0.74	1.29	0.18
	DICOM-MR	0.56	1.31	0.16	0.76	1.30	0.01
	3DPHM-MR	−0.29	1.14	1.00	0.03	1.29	0.18
3. Spatial relationship between the cardiac structures	DICOM-3DPHM	0.82	1.27	0.06	0.79	1.32	0.07
	DICOM-MR	0.85	1.28	0.10	1.15	1.02	0.00
	3DPHM-MR	0.03	1.22	1.00	0.35	1.12	1.00
4. Depth perception	DICOM-3DPHM	0.94	1.18	0.02	0.76	1.23	0.06
	DICOM-MR	1.09	1.16	0.00	1.09	1.16	0.00
	3DPHM-MR	0.15	1.13	0.66	0.32	1.17	0.40
5. Pathology learning	DICOM-3DPHM	0.85	1.28	0.02	0.62	1.35	0.26
	DICOM-MR	0.94	1.20	0.00	0.91	1.26	0.00
	3DPHM-MR	0.09	1.14	1.00	0.29	1.14	0.33
6. Communication tool with another health professional	DICOM-3DPHM	0.21	1.41	1.00	0.38	1.44	1.00
	DICOM-MR	−0.21	1.53	0.29	0.06	1.46	1.00
	3DPHM-MR	−0.41	1.26	0.29	−0.32	1.32	0.57
7. Communication tool with patients	DICOM-3DPHM	1.41	0.99	0.00	1.44	0.99	0.00
	DICOM-MR	0.35	1.04	0.29	0.50	0.93	0.12
	3DPHM-MR	−1.06	0.89	0.00	−0.94	1.01	0.01
8. Prepares me for surgery/intervention	DICOM-3DPHM	0.23	1.31	1.00	0.14	1.42	1.00
	DICOM-MR	0.45	1.60	0.57	0.68	1.36	0.23
	3DPHM-MR	0.23	1.31	1.00	0.55	1.26	0.34
9. Helps to understand possible complications	DICOM-3DPHM	0.30	1.33	1.00	0.39	1.41	1.00
	DICOM-MR	0.35	1.56	1.00	0.78	1.28	0.05
	3DPHM-MR	0.44	1.36	1.00	0.39	1.31	0.22
10. Pre-operative planning	DICOM-3DPHM	0.57	1.34	0.30	0.30	1.33	1.00
	DICOM-MR	0.74	1.36	0.00	0.87	1.32	0.02
	3DPHM-MR	0.17	1.30	1.00	0.57	1.24	0.22
11. Intra-operative guidance	DICOM-3DPHM	0.48	1.27	0.88	0.35	1.37	1.00
	DICOM-MR	0.70	1.43	0.36	0.83	1.30	0.14
	3DPHM-MR	0.22	1.35	1.00	0.18	1.27	0.08

^a^ after Bonferroni correction. 3DPHM, three-dimensional printed heart models; DICOM, Digital Imaging and Communications in Medicine; mean diff., mean difference; MR, mixed reality; SD, standard deviation.

**Table 4:** Mean differences in responses between interventionalists and non-interventionalists.

Questions ^a^	Mean Difference	*p*-Value
1. Assessment of major vessels	0.02	0.41
2. Appreciation of heart defects	−0.06	0.74
3. Spatial relationship between the cardiac structures	−0.01	0.50
4. Depth perception	0.26	0.66
5. Pathology learning	−0.15	0.85
6. Communication tool with another health professional	0.22	0.59
7. Communication tool with patients	0.14	0.86

^a^ only Question 1–7 were applicable for the analysis.

**Table 5:** Thematic analysis of qualitative data.

Themes	Feedbacks	Total
Intuitiveness of the clipping tool in the MR application	Relatively easy to use (*n* = 10) Not fully intuitive (*n* = 4) Steep learning curve (*n* = 2)	*n* = 16
Requirement of training for MR application	Training required to get the greatest benefit (*n* = 5) Training is needed (*n* = 4)	*n* = 9
Advantages of MR application	Clipping tool is very helpful in visualizing internal structures at different angles (*n* = 7) Help to plan surgeries (*n* = 2) Excellent 3D visualization (*n* = 3) Exciting possibilities to improve our practice (*n* = 1)	*n* = 13
Limitations of MR application	Creation of artificial defects from the clipping tool (*n* = 2) Difficult to look at structural connections (*n* = 1) Visual field of MR is too small (*n* = 1)	*n* = 4
Suggestions for MR application	A preset button to auto-crop the MR models (*n* = 2) Flat 2D ‘clipping plane’ is better (*n* = 1) Colored models (*n* = 1) Measuring tool (*n* = 1) Ability to offer ‘tunnel view’ (*n* = 1) Image definition needs improvement (*n* = 1) Ability to isolate the heart vessels or chambers (*n* = 1)	*n* = 8

2D, two-dimensional; 3D, three-dimensional; MR, mixed reality.

## Data Availability

The datasets used in this study are not publicly available due to restrictive requirements set out by authorized investigators.

## Supplementary Materials

The following supporting information can be downloaded at: https://www.mdpi.com/article/10.3390/biom12111548/s1, Supplementary File S1: The following supporting information can be downloaded at: https://youtu.be/WMVn6I-DKf0. This is a video link to demonstrate the mixed reality visualization of congenital heart disease. Supplementary File S2: Questionnaire used in the study. Supplementary File S3: Skewness and kurtosis for each question with regard to the three modalities. Supplementary File S4: Comparison of the mean rank difference of both CHD.

## References

[1] Biglino G., Koniordou D., Gasparini M., Capelli C., Leaver L.K., Khambadkone S., Schievano S., Taylor A.M., Wray J. (2017). Piloting the use of patient-specific cardiac models as a novel tool to facilitate communication during clinical consultations. Pediatr. Cardiol..

[2] Valverde I., Gomez-Ciriza G., Hussain T., Suarez-Mejias C., Velasco-Forte M.N., Byrne N., Ordoñez A., Gonzalez-Calle A., Anderson D., Hazekamp M.G. (2017). Three-dimensional printed models for surgical planning of complex congenital heart defects: An international multicentre study. Eur. J. Cardio-Thorac. Surg..

[3] Yoo S.J., Hussien N., Peel B., Coles J., van Arsdell G.S., Honjo O., Haller C., Lam C.Z., Seed M., Barron D. (2021). 3D modeling and printing in congenital heart surgery: Entering the stage of maturation. Front. Pediatr..

[4] Liang J., Zhao X., Pan G., Zhang G., Zhao D., Xu J., Li D., Lu B. (2022). Comparison of blood pool and myocardial 3D printing in the diagnosis of types of congenital heart disease. Sci. Rep..

[5] Hoashi T., Ichikawa H., Nakata T., Shimada M., Ozawa H., Higashida A., Kurosaki K., Kanzaki S., Shiraishi I. (2018). Utility of a super-flexible three-dimensional printed heart model in congenital heart surgery. Interact. CardioVascular Thorac. Surg..

[6] Olivieri L.J., Zurakowski D., Ramakrishnan K., Su L., Alfares F.A., Irwin M.R., Heichel J., Krieger A., Nath D.S. (2018). Novel, 3D display of heart models in the postoperative care setting improves CICU caregiver confidence. World J. Pediatr. Congenit. Heart Surg..

[7] Smith M., McGuinness J., O’Reilly M., Nolke L., Murray J., Jones J. (2017). The role of 3D printing in preoperative planning for heart transplantation in complex congenital heart disease. Ir. J. Med. Sci..

[8] Yang D.H., Park S.-H., Kim N., Choi E.S., Kwon B.S., Park C.S., Cha S.G., Baek J.S., Yu J.J., Kim Y.-H. (2021). Incremental value of 3D printing in the preoperative planning of complex congenital heart disease surgery. JACC Cardiovasc. Imaging.

[9] Loke Y.-H., Harahsheh A.S., Krieger A., Olivieri L.J. (2017). Usage of 3D models of tetralogy of Fallot for medical education: Impact on learning congenital heart disease. BMC Med. Educ..

[10] Hermsen J.L., Burke T.M., Seslar S.P., Owens D.S., Ripley B.A., Mokadam N.A., Verrier E.D. (2016). Scan, plan, print, practice, perform: Development and use of a patient-specific 3-dimensional printed model in adult cardiac surgery. J. Thorac. Cardiovasc. Surg..

[11] Sun Z., Lau I., Wong Y.H., Yeong C.H. (2019). Personalized three-dimensional printed models in congenital heart disease. J. Clin. Med..

[12] Lau I.W.W., Sun Z. (2019). Dimensional accuracy and clinical value of 3D printed models in congenital heart disease: A systematic review and meta-analysis. J. Clin. Med..

[13] Lau I., Gupta A., Sun Z. (2021). Clinical value of virtual reality versus 3D printing in congenital heart disease. Biomolecules.

[14] Sun Z. (2020). Clinical applications of patient-specific 3D printed models in cardiovascular disease: Current status and clinical applications. Biomolecules.

[15] Valverde I., Gomez G., Coserria J.F., Suarez-Mejias C., Uribe S., Sotelo J., Velasco M.N., Santos De Soto J., Hossienpour A.-R., Gomez-Cia T. (2015). 3D printed models for planning endovascular stenting in transverse aortic arch hypoplasia. Catheter. Cardiovasc. Interv..

[16] Valverde I., Gomez G., Byrne N., Anwar S., Silva Cerpa M.A., Talavera M.M., Pushparajah K., Velasco Forte M.N. (2022). Criss-cross heart three-dimensional printed models in medical education: A multicentre study on their value as a supporting tool to conventional imaging. Anat. Sci. Educ..

[17] Lau I., Sun Z. (2022). The role of 3D printed heart models in immediate and long-term knowledge retention in medical education. Rev. Cardiovasc. Med..

[18] Karkkainen J.M., Sandri G., Tenorio E.R., Alexander A., Bjellum K., Matsumoto J., Morris J., Mendes B.C., DeMartino R.R., Oderich G.S. (2019). Simulation of endovascular aortic repair using 3D printed abdominal aortic aneurysm model and fluid pump. Cardiovasc. Intervent. Radiol..

[19] Mitsuno D., Ueda K., Hirota Y., Ogino M. (2019). Effective application of mixed reality device holoLens: Simple manual alignment of surgical field and holograms. Plast. Reconstr. Surg..

[20] Moro C., Phelps C., Redmond P., Stromberga Z. (2021). HoloLens and mobile augmented reality in medical and health science education: A randomised controlled trial. Br. J. Educ. Technol..

[21] Gehrsitz P., Rompel O., Schöber M., Cesnjevar R., Purbojo A., Uder M., Dittrich S., Alkassar M. (2021). Cinematic rendering in mixed reality holograms: A new 3D preoperative planning tool in pediatric heart surgery. Front. Cardiovasc. Med..

[22] Soulami R.B., Verhoye J.-P., Nguyen Duc H., Castro M., Auffret V., Anselmi A., Haigron P., Ruggieri V.G. (2016). Computer-assisted transcatheter heart valve implantation in valve-in-valve procedures. Innovations.

[23] Opolski M.P., Artur D., Bartosz B., Staruch A.D., Kepka C., Rokicki J.K., Sieradzki B., Witkowski A. (2017). Feasibility and safety of augmented-reality glass for computed tomography-assisted percutaneous revascularization of coronary chronic total occlusion: A single center prospective pilot study. J. Cardiovasc. Comput. Tomogr..

[24] Microsoft HoloLens 2 Fundamentals: Develop Mixed Reality Applications. https://docs.microsoft.com/en-us/learn/paths/beginner-hololens-2-tutorials/.

[25] Ye W., Zhang X., Li T., Luo C., Yang L. (2021). Mixed-reality hologram for diagnosis and surgical planning of double outlet of the right ventricle: A pilot study. Clin. Radiol..

[26] Kumar R.P., Pelanis E., Bugge R., Brun H., Palomar R., Aghayan D.L., Fretland A.A., Edwin B., Elle O.J. (2020). Use of mixed reality for surgery planning: Assessment and development workflow. J. Biomed. Inform..

[27] Brun H., Bugge R.A.B., Suther L.K.R., Birkeland S., Kumar R., Pelanis E., Elle O.J. (2019). Mixed reality holograms for heart surgery planning: First user experience in congenital heart disease. Eur. Heart J. Cardiovasc. Imaging.

[28] Microsoft Spatial awareness getting started—MRTK2. 8 March 2022. https://docs.microsoft.com/en-us/windows/mixed-reality/mrtk-unity/mrtk2/features/spatial-awareness/spatial-awareness-getting-started?view=mrtkunity-2022-05.

[29] Wu C.A., Squelch A., Sun Z. (2021). Investigation of three-dimensional printing materials for printing aorta model replicating type B aortic dissection. Curr. Med. Imaging.

[30] Wu C.A., Squelch A., Jansen S., Sun Z. (2021). Optimization of computed tomography angiography protocols for follow-up type B aortic dissection patients by using 3D printed model. Appl. Sci..

[31] Sun Z., Wee C. (2022). 3D printed models in cardiovascular disease: An exciting future to deliver personalized medicine. Micromachines.

[32] Kaufmann R., Zech C.J., Takes M., Brantner P., Thieringer F., Dentschmann M., Hergan K., Scharinger B., Hecht S., Rezar R. (2022). Vascular 3D printing with a novel biological tissue mimicking resin for patient-specific procedure simulations in interventional radiology: A feasibility study. J. Digit. Imaging.

[33] Lee S., Squelch A., Sun Z. (2021). Quantitative assessment of 3D printed model accuracy in delineating congenital heart disease. Biomolecules.

[34] Lau I.W.W., Liu D., Xu L., Fan Z., Sun Z. (2018). Clinical value of patient-specific three-dimensional printing of congenital heart disease: Quantitative and qualitative assessments. PLoS ONE.

[35] Yoo S.J., Spray T., Austin E.H., Yun T.J., van Arsdell G.S. (2017). Hands-on surgical training of congenital heart surgery suing 3-dimensional print models. J. Thorac. Cardiovasc. Surg..

[36] Brunner B.S., Thierij A., Jakob A., Tengler A., Grab M., Thierfelder N., Leuner C.J., Haas N.A., Hopfner C. (2022). 3D-printed heart models for hands-on training in pediatric cardiology-the future of modern learning and teaching?. GMS J. Med. Educ..

[37] Newe A., Becker L. (2018). Three-dimensional portable document format (3D PDF) in clinical communication and biomedical sciences: Systematic review of applications, tools and protocols. JMIR Med. Inform..

[38] Newe A., Becker L., Schenk A. (2014). Application and evaluation of interactive 3D PDF for presenting and sharing planning results for liver surgery in clinical routine. PLoS ONE.

