# Supplementary material for: Clinical Applications of Mixed Reality and 3D Printing in Congenital Heart Disease
Source: Biomolecules. 2022 Oct 24;12(11):1548. doi: 10.3390/biom12111548 (PMC9687840; doi:10.3390/biom12111548)
Supplement: Supplementary file 1 [file biomolecules-12-01548-s001.zip › Supplementary Files S2¿CS4/Supplementary File S2.pdf]

SURVEY: Cardiac surgeons, Interventional cardiologists

Age	
Gender	
Medical Specialty	☐ Cardiac surgeon ☐ Interventional cardiologist
Have you ever used AR before?	☐ Yes ☐ No
Have you ever used 3D printing technology in your specialty?	☐ Yes ☐ No

Please rank the following with 1 being the highest/ best, and 3 being the lowest/ worst.

	**ATRIAL SEPTAL DEFECT**			**DOUBLE OUTLET RT VENTRICLE**		
**Questions**	DICOM images	3D printed models	HoloLens 2	DICOM images	3D printed models	HoloLens 2
1. The major vessels of the heart can be well assessed.						
2. The heart defects can be easily appreciated.						
3. The spatial relationship between the cardiac structures can be easily understood.						
4. The model offers a good depth perception for the heart structures.						
5. I prefer the use of this method to learn about the pathology.						
6. I prefer the use of this method to communicate with another health professional during discussion.						
7. I prefer the use of this method to communicate with patients about the pathology.						
8. This model prepares me for surgery.						
9. This model helps me to understand the possible complications related to the procedure/ surgery.						
10. I am likely to consult this model for preoperative planning.						
11. This model can be helpful for intraoperative guidance.						

12. How do you feel about the ability to change the clipping plane on the AR models?

13. Additional comments:

SURVEY: Radiologists, doctors, junior doctors

Age	
Gender	
Medical Specialty & Year of Experience	
Have you ever used AR before?	☐ Yes ☐ No
Have you ever used 3D printing technology in your specialty?	☐ Yes ☐ No

Please rank the following with 1 being the highest/ best, and 3 being the lowest/ worst.

	**ATRIAL SEPTAL DEFECT**			**DOUBLE OUTLET RT VENTRICLE**		
**Questions**	DICOM images	3D printed models	HoloLens 2	DICOM images	3D printed models	HoloLens 2
1. The major vessels of the heart can be well assessed.						
2. The heart defects can be easily appreciated.						
3. The spatial relationship between the cardiac structures can be easily understood.						
4. The model offers a good depth perception for the heart structures.						
5. I prefer the use of this method to learn about the pathology.						
6. I prefer the use of this method to communicate with another health professional during discussion.						
7. I prefer the use of this method to communicate with patients about the pathology.						

8. How do you feel about the ability to change the clipping plane on the AR models?

9. Additional comments:
