# Supplementary material for: Clinical Applications of Mixed Reality and 3D Printing in Congenital Heart Disease
Source: Biomolecules. 2022 Oct 24;12(11):1548. doi: 10.3390/biom12111548 (PMC9687840; doi:10.3390/biom12111548)
Supplement: Supplementary file 1 [file biomolecules-12-01548-s001.zip › Supplementary Files S2¿CS4/Supplementary File S3.pdf]

Supplementary File S3:

Table: Skewness and kurtosis for each question

Question	Modality	Atrial Septal Defect		Double Outlet Right Ventricle	
		Skewness	Kurtosis	Skewness	Kurtosis
1. Assessment of major vessels	DICOM	0.297	-1.595	-0.181	-1.787
	3DP	-0.034	-0.651	-0.078	-0.804
	MR	-0.244	-1.809	0.297	-1.595
2. Appreciation of heart defects	DICOM	-1.104	-0.570	-1.151	-0.156
	3DP	0.764	-0.722	0.414	-1.026
	MR	0.100	-0.625	0.487	-1.032
3. Spatial relationship between the cardiac structures	DICOM	-1.375	0.329	-1.737	1.572
	3DP	0.435	-0.862	0.248	-1.101
	MR	0.513	-0.865	0.538	-0.735
4. Depth perception	DICOM	-1.897	2.106	-1.589	1.121
	3DP	0.354	-0.678	0.213	-0.867
	MR	0.677	-0.489	0.884	-0.247
5. Pathology learning	DICOM	-1.497	0.637	-1.184	-0.267
	3DP	0.435	-0.862	0.186	-1.027
	MR	0.486	-0.592	0.677	-0.489
6. Communication tool with another health professional	DICOM	0.000	-1.771	-0.297	-1.595
	3DP	0.344	-0.997	0.449	-1.194
	MR	-0.403	-1.343	-0.162	-1.368
7. Communication tool with patients	DICOM	-1.214	0.556	-1.233	0.638
	3DP	2.986	8.127	2.728	6.050
	MR	0.079	-0.144	0.067	0.253
8. Prepares me for surgery / intervention	DICOM	-0.485	-1.532	-0.574	-1.282
	3DP	0.000	-0.685	-0.249	-1.225
	MR	0.485	-1.532	0.847	-0.538
9. Helps to understand possible complications	DICOM	-0.454	-1.480	-0.851	-0.765
	3DP	0.139	-1.008	0.000	-1.392
	MR	0.269	-1.659	0.773	-0.587
10. Pre-operative planning	DICOM	-0.988	-0.578	-0.851	-0.765
	3DP	0.228	-1.140	-0.139	-1.008
	MR	0.601	-0.974	1.068	-0.168
11. Intra-operative guidance	DICOM	-0.851	-0.765	-0.851	-0.765
	3DP	0.139	-1.008	-0.076	-1.223
	MR	0.647	-1.190	0.916	-0.414

3DP, three-dimensional printing; DICOM, Digital Imaging and Communications in Medicine; MR, mixed reality
