# Supplementary material for: Clinical Applications of Mixed Reality and 3D Printing in Congenital Heart Disease
Source: Biomolecules. 2022 Oct 24;12(11):1548. doi: 10.3390/biom12111548 (PMC9687840; doi:10.3390/biom12111548)
Supplement: Supplementary file 1 [file biomolecules-12-01548-s001.zip › Supplementary Files S2¿CS4/Supplementary File S4.pdf]

Supplementary File S4:

Table: Comparison of the mean ranks difference of both CHD

Question	Modality	Mean difference (ASD-DORV)	p-value[a]
1. Assessment of major vessels	DICOM	-0.24	0.26
	3DP	-0.03	0.44
	MR	0.27	0.16
2. Appreciation of heart defects	DICOM	-0.03	0.72
	3DP	-0.14	0.65
	MR	0.17	0.43
3. Spatial relationship between the cardiac structures	DICOM	-0.09	0.50
	3DP	-0.11	0.55
	MR	0.21	0.39
4. Depth perception	DICOM	0.06	0.68
	3DP	-0.11	0.42
	MR	0.06	0.35
5. Pathology learning	DICOM	0.09	0.82
	3DP	-0.14	0.25
	MR	0.06	0.32
6. Communication tool with another health professional	DICOM	-0.15	0.01
	3DP	0.03	0.44
	MR	0.12	0.11
7. Communication tool with patients	DICOM	-0.06	0.09
	3DP	-0.03	0.77
	MR	0.09	0.22
8. Prepares me for surgery / intervention	DICOM	-0.04	0.88
	3DP	-0.14	0.78
	MR	0.18	0.80
9. Helps to understand possible complications	DICOM	-0.17	0.02
	3DP	-0.09	0.79
	MR	0.26	0.09
10. Pre-operative planning	DICOM	0.04	0.74
	3DP	0.10	0.09
	MR	0.18	0.42
11. Intra-operative guidance	DICOM	0.00	0.45
	3DP	-0.13	0.23
	MR	0.13	0.17

[a] after Bonferroni correction

3DP, three-dimensional printing; DICOM, Digital Imaging and Communications in Medicine; MR, mixed reality
